# Incidence of oral manifestations in hematological malignancy patients undergoing chemotherapy: prospective cohort study

**DOI:** 10.4317/medoral.26652

**Published:** 2024-12-24

**Authors:** Estthelamares Lúcio da Silva Mello, Nayara Gabriela Silva Pena, Virginia Andrade de Souza, Camila Maria da Silva, Lucas Nascimento Ribeiro, Raylane Farias de Albuquerque, Marco Meleti, Paulo Vescovi, Jair Carneiro Leão, Igor Henrique Morais Silva

**Affiliations:** 1 Residents of the Dentistry Department at the Hospital do Cancer de Pernambuco, Recife, PE, Brazil; 2 Department of Dentistry, Hospital do Cancer de Pernambuco, Recife, PE, Brazil; 3 Department of Surgery and Medicine, Università di Parma, Parma, Italy; 4 Department of Clinics and Preventive Dentistry, Universidade Federal de Pernambuco

## Abstract

**Background:**

Oral manifestations are frequent in patients with hematological malignancies undergoing chemotherapy and may be directly or indirectly related to drug toxicity. Few studies have assessed the chemotherapy and oral manifestations that patients might develop. Therefore, this study aimed to evaluate the incidence of oral manifestations in patients with hematologic neoplasms during hospitalization and chemotherapy.

**Material and Methods:**

A Prospective Cohort Study, in which adult patients diagnosed with hematological malignancies undergoing chemotherapy were followed up daily to observe the possible development of oral manifestations. Sociodemographic and oncological data as well as oral manifestations when presented were collected. All the patients underwent photobiomodulation to prevent mucositis. STATA/SE 12.0, and Excel 365 software were used to assess the collected data. Fisher’s exact test was used to assess categorical variables.

**Results:**

95 patients were included in this study. Sixty four (67,4%) of the patients were male, 31 female (32,6%) and the mean age was 44 years of age (SD 15,1) ranging from 19 to 79. Non-Hodgkin’s lymphoma is the most common neoplasm. Cytarabine was the most commonly administered drug used in 72 patients (75,8%). Fungal infections were the most frequent oral manifestations. There was no correlation between the histological diagnosis and the risk of developing oral mucositis.

**Conclusions:**

Oral manifestations are frequent in patients with hematological malignancies who are undergoing chemotherapy. Since there is a tendency for increasing cases of hematologic neoplasms annually, it is of paramount importance to know the treatments offered to these patients and the related side effects in order to prevent them and consequently decrease morbimortality.

** Key words:**Oncology, oral mucosa, antineoplastics.

## Introduction

Dental care for oncological patients is demanding and should be initiated before the initiation of oncological treatment (1,2). It is well known that following the patient before and during the oncological treatment has as benefit the significant reduction of the risks of developing lesions in the oral cavity (3,4).

Among the most common hematologic neoplasms, there are three significant groups: Leukemia, Lymphoma with subtypes, and Multiple Myeloma. The available treatments for these neoplasms include chemotherapy, immunotherapy, radiotherapy, and hematopoietic stem cell transplantation. Chemotherapy is the most common treatment. There is a specific protocol for each neoplasm and its subtype. Each protocol includes different classes of drugs, and each drug acts at different cell sites (5-7).

Opposing to targeting therapy, chemotherapy operates in a non-specific way, therefore, cytotoxic effects may affect normal cells with high proliferative rate such as the cells of the gastrointestinal system, including the oral cavity (4,8)

Changes in the oral cavity may be due to the direct cytotoxicity of chemotherapy or indirectly due to myelosuppression (7,9). Oral mucositis, fungal and viral infections, xerostomia, vascular disturbance such as haemorrhagia, haematoma, petechiae; trismus and abscess can be observed in patients under chemotherapy (7,10). These manifestations can lead to secondary effects such as fever, bacteremia, dysgeusia, and dysphagia. Therefore all of these complications should be prevented and treated as soon as possible, since they can increase the risk of morbimortality in immunosuppressed patients (7,9,10).

Few studies have aimed to evaluate the frequency of oral manifestations in hematological patients and the chemotherapy protocols to which these patients have been submitted. Some complications related to chemotherapy are already known; however, the direct effect of each drug class and protocol, the intensity at which they act, or the dose and route of administration present higher risks of developing lesions in the oral cavity, which is not well known (6).

Thus, this study aimed to evaluate the incidence of lesions in the oral cavity in patients with hematological malignancies during hospitalization for chemotherapy by identifying oral manifestations, diagnosis, and graduate cases of oral mucositis.

## Material and Methods

This prospective cohort study was conducted in the oncohematology ward of the Hospital of Cancer of Pernambuco.

Patients with a histological diagnosis of hematological malignancy who were hospitalized to be submitted to chemotherapy protocols with or without target therapy with rituximab regardless of the cycle were included in this study. Patients who were unable to answer the questionnaire or those for whom it was not possible to examine the oral cavity were ineligible for the study.

The research was submitted to the Hospital of Cancer of Pernambuco’s Ethics Committee for Research with Human Beings, as well as the necessary terms, respecting the legal and ethical aspects, according to the Resolution 466/2012, the research only started after the Ethics Committee approval. Number of approval: 60449422.7.0000.5205.

The patients were clarified regarding the content of the research, and after agreeing to be included in it, they signed the Informed Consent Form, where a copy of it remained with the patients and another with the research team.

The collected data were registered in the patient file, which was elaborated by the main research and consisted of sociodemographic, oncological diagnosis, chemotherapy (protocol, drugs, and route of administration), hospitalization period, manifestations in the oral cavity, and oral mucositis grading (when present) data. The patients were evaluated on a daily basis and submitted to low-level laser therapy (LLLT) from the first day of chemotherapy until hospital discharge, except on the weekends when there was no dental service. All patients were submitted to LLLT, regardless of presence or absence of oral mucositis. The ones without any oral signs of mucositis were submitted to LLLT in order to prevent this oral manifestation, and the patients already presenting signs of oral mucositis on the first day of chemotherapy were submitted to LLLT in order to treat this oral manifestation.

Collected data regarding oral manifestations included the presence or absence of oral mucositis, fungal infections (candidiasis, stomatitis, and angular cheilitis), viral infections (simplex herpes), xerostomia, hyposalivation, vascular disturbances (hematoma and petechiae), and necrotizing ulcerative gingivitis during or after chemotherapy.

All oral manifestations were diagnosed exclusively through oral examination performed with a lantern (Ecleris, San Diego, United States) and a wooden palette. The responsible researchers proceeded to reduce any pain or discomfort that the patient might feel during the oral cavity inspection.

Oral mucositis was graded according to the World Health Organization Mucositis Oral Toxicity Scale, where grade 0 is the absence of alterations in the oral cavity; grade 1 is the presence of pain and erythema; grade is the 2 presence of erythema and ulcers; grade 3 is the presence of ulcers and the patient can only feed through a liquid diet; and grade 4, the patient is unable to feed.

All patients undergoing chemotherapy underwent LLLT regardless of the protocol and cycle. Diode laser, DMC Therapy XT (DMC, São Paulo, Brazil), with a 660 nm wave-lenght (red laser) was used. LLLT was performed at the following predetermined sites in the oral cavity: three spots in the buccal mucosa bilaterally, three spots in the upper and lower lip, three spots in the lateral border of the tongue bilaterally, two spots in the dorsum of the tongue following the midline until reaching the frenulum, and three spots on the floor of the mouth bilaterally.

The dosimetric characteristics were: 100mW, 1 J/spot, 10 s/spot, and 35 J/cm ², respectively. A silica optical fiber with a length of 10 cm and spot of 0.028 cm ² was used as the optical conductor.

For oral cavity evaluation and LLLT application, all the researchers were trained and followed a predetermined protocol to reduce the risk of bias.

Alcohol at 70% was used as a disinfection method for the laser device, and a plastic barrier was used for LLLT in each patient, who was discharged after use. During the laser therapy, the patient was requested to use specific safety glasses. The patients underwent LLLT during hospitalization.

STATA/SE 12.0 and Excel 365 software were used to evaluate the collected data. Fisher's exact test was used to assess categorical variables. All tests were applied with 95% confidence, and all results were calculated considering valid responses, that is, ignored responses were not accounted for.

The numerical variables are represented by measures of the central tendency of dispersion. The results are presented in a Table along with their respective absolute and relative frequencies.

## Results

A total of 95 chemotherapy cycles were included in this study. Of these, 67,4% of the patients were male and 32,6% were female. The mean age of the patients was 44 years.

Non-Hodgkin’s lymphoma was the most common histological diagnosis observed, accounting for 52,6% of the cases, while Multiple Myeloma was the least frequent, accounting for only 1,1% of the cases.

The mean hospitalization time was 24,89 days with a maximum period of hospitalization of 115 days and minimum of 3 days.

Among the protocols observed, cytarabine was the most frequent drug present in 75,8% of the chemotherapies, followed by methotrexate, which accounted for 44,2% of the protocols.

The sociodemographic and oncological data are presented in Table 1. Data regarding patient's age and hospitalization period can be seen in Table 2.

The most common route used for protocol administration was intravenous administration, present in 62,1% of the cases, followed by intravenous administration associated with intrathecal (33,7%). The least frequent routes of administration were intravenously associated with intramuscular and subcutaneous administration, both of which were present in only 2,1% of cases.

Fungal infections were the most common oral manifestations observed, followed by oral mucositis, which when present was not severe, most cases were graded into I or II.

Oral mucositis, Necrotizing Ulcerative Gingivitis (NUG) and herpes were observed at a higher frequency after the protocol administration. Candidiasis, stomatitis, and angular cheilitis were more frequently observed during chemotherapy.

All the manifestations observed and their frequencies in terms of percentages are shown in Table 3.

We investigated whether patients’ diagnoses were related to a higher risk of developing oral mucositis. Nevertheless, no statistically significant associations were observed between these variables. The detailed crossing data are listed in Table 4.

## Discussion

The incidence of hematological neoplasms varies according to age, sex, and socioeconomic status of the individual (11). In the present study, a higher prevalence of male patients was observed, which was also observed in similar study (12) and according to Zhang *et al*. (13) in their review on global hematologic neoplasm prevalence.

) The review performed by Zhang *et al*. (13) shows that there is a higher incidence of non-Hodgkin lymphoma, Hodgkin lymphoma, and leukemia in male patients. Data from Horesh and Horowitz study (14) show that, globally, Non Hodgkin Lymphoma has a higher incidence in male individuals.

Only adult patients were included in this study. The mean patient age was 44 years, and the most common neoplasm was Non-Hodgkin Lymphoma. In a study by Nishi *et al*. (12), the most frequent neoplasm observed was lymphoma, followed by leukemia, and multiple myeloma; these results are similar to ours. However, different results have been reported in the literature. In Chen *et al*. (10) study, Acute Myeloid Leukemia was the most prevalent neoplasm, meanwhile in the Ramirez-Amadir *et al*. (7) Leukemia was the most commonly reported neoplasm. The location where the data were surveyed and the methodology applied may explain the difference between our results and those reported in the literature.

Data from Thandra *et al*. (15) show that Non-Hodgkin Lymphoma is commonly diagnosed in patients between 39 and 65 years of age. This age group is similar to that observed in the present study.

Oral manifestations in patients undergoing chemotherapy are common; Ramirez-Amador *et al*. (7) reported that 45% of patients develop manifestations in the oral cavity. This percentage varies in the literature, as reported by Jena *et al*. (4), in which 60% of the patients presented with one or more oral manifestations.

In the present study, fungal infections (candidiasis, stomatitis, and angular cheilitis), followed by oral mucositis, were the most common manifestations in the oral cavity of patients. More than half of the patients in Chen *et al*. ’s study (10) presented with infections in the oral cavity, and *Candida* Albicans was the most frequent pathogen related to these infections. According to literature, manifestations in the oral cavity may vary. In a study by Ramirez-Amador *et al*. (7), exfoliative cheilitis, hemorrhages, and herpes infections were the most common oral manifestations and were more prevalent than oral mucositis. However, in studies by García-Chías *et al*. (16) and Jena *et al*. (4), xerostomia was the most frequent complication observed, covering more than 70% of the cases in the previous study. Only 5 cases of xerostomia were observed in the present study.

As seen in the study of García-Chías *et al*. (16) patients undergoing oncological treatment can be polypharmacy, and use drugs to treat complications in the cardiovascular and gastrointestinal systems or another systemic disease. This may be related to a higher risk of xerostomia development, as well as the chemotherapeutic regimens the patient is being submitted (17).

Herpes infections were not a common finding in our study, in contrast to other reports in the literature (7). Chen *et al*. (10) study concluded that antifungal prophylaxis is correlated with a higher risk of developing viral infections, such as simplex herpes, resulting from changes in the oral microbiota. Thus, prophylaxis is ideal for both types of infections. At the Hospital of Cancer of Pernambuco, the adopted protocol is to provide prophylaxis for fungal and viral infections, which could explain why few cases of viral infections were observed in our study. However, it is worth noting that in the present study, the diagnosis was made only through clinical evaluation, with no swabs or cultures of infectious lesions performed, which could also explain the fewer number of cases of herpetic lesions observed.

Sixteen % of the oral mucositis cases in this study were observed in the periods after chemotherapy administration; in contrast, only 6,3% of the oral mucositis cases were observed during the chemotherapy administration period. The first signs of oral mucositis can be seen around 4 days after chemotherapy (18), and may persist for 3 weeks, reaching a peak around 7 and 14 days after administration (19). This explains why the majority of oral mucositis cases were observed after chemotherapy administration.

A large percentage of the cases of oral mucositis are classified as grade I or II, and there are few records of severe grades of mucositis. An integrative review carried out by Curra *et al*. (6) observed that patients with hematologic neoplasms undergoing chemotherapy may have severe mucositis. Regardless of the cycle and protocol, all the patients in our study underwent photobiomodulation. Therefore, the risk of developing severe mucositis was reduced, as there is consensus in the literature that low-intensity laser therapy acts prophylactically for oral mucositis (20,21), which may explain the low number of severe cases.

Regarding the drugs and protocols, there may be an incidence of oral mucositis in patients using daunorubicin and etoposide, as well as in patients using cytarabine at high doses and methotrexate. Protocols such as CHOP (cyclophosphamide, doxorubicin, vincristine, and prednisone) are also associated with a high rate of severe oral mucositis (6). Such drugs were frequently observed in the chemotherapy protocols administered to patients included in the study. Rituximab, a target therapy, was observed in 18% of all protocols included in the present study. In the literature there are case reports of the development of oral lichenoid reaction secondary to Rituximab treatment (22,23), these oral modifications were not observed in our patients. A laboratory study performed in rats demonstrated that administration of this anti-CD20 monoclonal antibody delayed the healing process of oral induced traumatic ulcers due to its reduction of inflammatory cell migration (24). Due to the small sample we could not cross the data between protocols with and without Rituximab in order to confirm the delay in oral mucositis healing. Therefore, studies focusing primarily on the relationship should be performed.

Chen *et al*. (10) found that among hematologic malignancies, those of myeloid origin were associated with an increased risk of developing oral mucositis. It was observed that myeloid neoplasms have an almost 5x greater risk of developing oral mucositis than patients diagnosed with lymphoid neoplasms, and 10x greater than those diagnosed with multiple myeloma. It is worth noting that there were numerous related variables. For example, it is known that side effects in the oral cavity are associated with the specific phase of treatment that the patient is undergoing. Patients undergoing chemotherapy in the maintenance and consolidation phases have a higher risk of presenting with manifestations in the oral cavity than those in the induction phase (25).

In conclusion, this study explored the incidence of oral manifestations in patients undergoing chemotherapy for hematological malignancies with a particular focus on the presence of oral mucositis. Despite thorough examination and analysis, our findings did not reveal a statistically significant association between patients' histological diagnoses and the development of oral mucositis. Although this outcome may initially appear surprising, it underscores the complexity of the interplay between various factors contributing to oral mucositis in this patient population. Moreover, it highlights the need for further exploration of additional variables, such as treatment protocols, patient-specific characteristics, and underlying biological mechanisms, to comprehensively understand the multifaceted nature of oral manifestations in hematological malignancies. Such insights are crucial for refining therapeutic approaches and improving the overall management of oral complications in these vulnerable patients, ultimately enhancing their quality of life throughout the course of their treatment. Additionally, according to data from INCA and Globocan, the number of cases of hematological malignancies, especially NHL, is increasing, emphasizing the importance of ongoing research efforts to address the evolving challenges faced by patients with these conditions.

## Figures and Tables

**Table 1:** Patients’ sociodemographic and oncologic data.

Variables		n	%
Sex	Female	31	32,6
	Male	64	67,4
Race	White	28	29,8
	Black	66	70,2
Histological diagnosis	Non Hodgkin Lymphoma (NHL)	50	52,6
	Acute Lymphoid Leukemia (ALL)	10	10,5
	Acute Myeloid Leukemia (AML)	21	22,1
	Adult T-cell leukemia/lymphoma	4	4,2
	Hodgkin Lymphoma (HL)	7	7,4
	Multiple Myeloma (MM)	1	1,1
	Chronic Lymphocytic Leukemia (CLL)	2	2,1
Chemotherapy drugs	Methotrexate	42	44,2
	Cytarabine	72	75,8
	Etoposide	14	14,7
	Cyclophosphamide	28	29,5
	Doxorubicin	23	24,2
	Vincristine	28	29,5
	Rituximab	18	18,9
	Fludarabine	2	2,1
	Daunorubicin	12	12,6
	Carboplatin	5	5,3

**Table 2:** Patient's age and hospitalization period data.

Variable	Mean ± DP	Median (P_25_; P_75_)	Min. - Max.
Age	44,0 ± 15,1	46,0 (32,0; 56,0)	19,0 - 79,0
Hospitalization period	24,9 ± 20,3	19,0 (14,0; 28,0)	3,0 - 115,0

**Table 3:** Oral manifestations observed in the hospitalized patients during and after chemotherapy protocol administration.

Variables		n	%
Oral mucositis during	Yes	6	6,3
	No	89	93,7
Oral mucositis after	Yes	16	16,8
	No	79	83,2
Oral mucositis grading	Grade 1	8	44,4
	Grade 2	8	44,4
	Grade 3	1	5,6
	Grade 4	1	5,6
Oral manifestations	Xerostomia during	4	4,2
	Xerostomia after	1	1,1
	Hyposalivation during	3	3,2
	Hyposalivation after	1	1,1
	Candidiasis/stomatitis during	9	9,5
	Candidiasis/stomatitis after	7	7,4
	Cheilitis during	5	5,3
	Cheilitis after	3	3,2
	Herpes during	0	0,0
	Herpes after	2	2,1
	Petechiae during	3	3,2
	Petechiae after	3	3,2
	NUG during	0	0,0
	NUG after	1	1,1

**Table 4:** Histological diagnosis and risk of oral mucositis development during and after chemotherapy administration.

Variables		Histologic diagnosis			*p-value* *
		NHL	ALL	AML	
		n (%)	n (%)	n (%)	
Mucositis during	Yes	3 (6,0)	2 (20,0)	1 (4,8)	0,292
	No	47 (94,0)	8 (80,0)	20 (95,2)	
Mucositis After	Yes	12 (24,0)	0 (0,0)	2 (9,5)	0,128
	No	38 (76,0)	10 (100,0)	19 (90,5)	

(*) Fisher's Exact Test.
